# ZnNPs and senps co-adjuvants deliver superior and long-lasting protection: first report in inactivated Rift Valley fever vaccines for sheep

**DOI:** 10.1007/s11250-025-04783-z

**Published:** 2025-12-24

**Authors:** Sally Zaki Abdelrahman Hafez, Adel Abdelazim Fayed, Taradi Abdelfattah Sayed, Amany Dieb Bahr, Mohamed S. Kamel

**Affiliations:** 1 Rift Valley Fever Department, Vet. Serum and Vaccine Research Institute, Abbasia, Cairo, Egypt; 2 Department of Medicine and Infectious Diseases, Faculty of Veterinary Medicine, Cairo University, Giza, Egypt

**Keywords:** Rift Valley fever, Nanoparticle adjuvants, Zinc oxide nanoparticles (ZnNPs), Selenium nanoparticles (SeNPs), Immunogenicity, Protective immunity

## Abstract

Rift Valley Fever (RVF) poses a significant threat to livestock and human health, necessitating effective and safe vaccines. This study evaluated the immunogenicity and safety of novel inactivated RVF vaccines in sheep, enhanced with zinc oxide nanoparticles (ZnNPs) and selenium nanoparticles (SeNPs) as co-adjuvants, compared to an aluminum hydroxide gel (ALHV) adjuvanted vaccine alone. Our findings demonstrate that nanoparticle-formulated ALHV elicited a markedly faster onset of protection, achieving protective neutralizing antibody titers within 3–4 days post-vaccination, significantly accelerating immunity compared to approximately two weeks with ALHV alone. Furthermore, these enhanced formulations induced more robust and sustained humoral and IFN-γ responses, indicating superior activation of both antibody-mediated and cell-mediated immunity. The nanoparticle-enhanced vaccines also maintained long-lasting protection, extending the duration of protective antibody titers up to 11 months, compared to 10 months for ALHV alone. Beyond immunological benefits, these vaccines were safe, stable, and associated with improved growth performance in vaccinated sheep, particularly with ZnNPs. Biochemical analyses confirmed the absence of adverse effects on liver and kidney function, while showing beneficial increases in total protein and globulin levels. This study underscores the potential of SeNPs and ZnNPs as potent adjuvants for inactivated RVF vaccines, offering a promising strategy for developing more effective, rapidly protective, and durable vaccines against RVF and potentially other zoonotic diseases.

## Introduction

Rift Valley fever virus (RVFV), a member of the genus *Phlebovirus* within the *Bunyaviridae* family and the order *Bunyavirales*, is the causative agent of Rift Valley fever (RVF), a zoonotic disease that significantly impacts both animal and human health. This virus poses a substantial clinical and economic burden, particularly in wild and domestic ruminants, such as sheep, goats, cattle, and camels. Transmission to humans occurs through direct contact with contaminated animal products or via arthropod vectors, leading to a range of clinical manifestations, including severe complications such as hemorrhagic fever and encephalitis (Mohapatra et al. 2023; Kitandwe et al. 2024). In livestock, RVFV outbreaks are often devastating, resulting in high fetal and neonatal mortality rates, further amplifying its socio-economic impact on agriculture and food security (Petrova et al. 2020).

Initially confined to Africa, RVFV has since demonstrated potential for widespread dissemination. Outbreaks have now been reported in regions such as Madagascar, the Comoros Islands, Saudi Arabia, and Yemen (Ikegami 2012; Samy et al. 2017). This expansion is attributed to the wide distribution of competent vectors in previously non-endemic areas, a phenomenon that is expected to intensify due to global climate change (Faburay et al. 2017). Consequently, RVFV presents a growing transboundary threat, making effective control measures an urgent priority.

Vaccination remains one of the most effective tools for controlling infectious diseases, including RVF. However, current RVF vaccines are associated with critical limitations. For instance, the Smithburn live-attenuated vaccine, while providing robust protection, is unsuitable for use in pregnant animals due to safety concerns (Botros et al. 2006; Kamal 2009). In contrast, the inactivated whole-virus vaccine is safer and can be used across all physiological stages, but its production is costly, and multiple booster doses are required to achieve optimal protection (Matsiela et al. 2023). Additionally, the recently developed Clone 13 vaccine has shown promising efficacy and safety in preclinical trials conducted on sheep and cattle. However, its effectiveness and safety under field conditions have not been thoroughly documented (Dungu et al. 2010). Given these challenges, there is an urgent need for next-generation RVF vaccines that are both safe and cost-effective, while eliciting durable immunity.

To address these limitations, advancements in vaccine adjuvant technologies offer a promising path forward. Adjuvants, as essential components in vaccine formulations, are substances specifically designed to enhance the body’s immune response to the vaccine and improve its overall effectiveness. Conventional adjuvants, such as aluminum hydroxide (alum), have been widely used to amplify antigen-specific immunity; however, their effects are limited in scope (Barnowski et al. 2019). Recent developments in nanotechnology have introduced a new class of adjuvants that provide unique advantages over traditional formulations. These nanomaterials not only improve antigen delivery through enhanced bioavailability and controlled release, but also possess intrinsic immunomodulatory properties that can shape and amplify immune responses in a targeted manner (Zhu et al. 2014).

Among these, metal-based nanoparticles, such as zinc oxide (ZnNPs) and selenium nanoparticles (SeNPs), have emerged as particularly promising candidates for vaccine adjuvants. ZnNPs are valued for their biocompatibility, cost-effectiveness, and ability to enhance immune responses. Studies have demonstrated that ZnNPs significantly increase the production of antigen-specific antibodies, with evidence suggesting that they promote a Th2-skewed immune response (Roy et al. 2014). Furthermore, their structural stability and potential for large-scale production make them highly suitable for vaccine applications (Sharma et al. 2019).

Similarly, SeNPs have garnered attention due to their low toxicity and potent immunomodulatory effects. These nanoparticles have been shown to enhance both innate and adaptive immunity by stimulating T cells, natural killer (NK) cells, and other immune pathways (Mahdavi et al. 2017; Ranjbariyan et al. 2022). Recent studies suggest that SeNP-based adjuvants can promote Th1-skewed immunity and generate high-titer antigen-specific neutralizing antibodies, making them particularly effective in combating viral infections (Lai et al. 2023). Additionally, their ability to retain the beneficial properties of selenium ions while exhibiting reduced toxicity makes SeNPs a safer alternative for biomedical applications (Shakibaie et al. 2012).

Leveraging the unique properties of these nanomaterials, this study aims to develop an inactivated RVF vaccine that incorporates alum hydroxide gel as a primary adjuvant, with zinc oxide and selenium nanoparticles serving as co-adjuvants. By combining the proven safety of traditional adjuvants with the immunomodulatory advantages of nanotechnology, this approach aims to overcome the limitations of existing vaccines. The newly developed vaccine will be evaluated for its efficacy, immunogenicity, and duration of the immune response, with a direct comparison to the currently available inactivated RVF vaccine in South Africa. Ultimately, this study seeks to advance the development of a next-generation RVF vaccine that is safe, effective, and suitable for deployment in both endemic and non-endemic regions.

## Materials and methods

### Virus

The RVFV, ZH501 strain, was used in this study. The virus was provided by the RVF Vaccine Research Department at the Veterinary Serum and Vaccine Research Institute (VSVRI), Abbasia, Cairo, Egypt, and was prepared following the methods described by Grigorov et al. 2011. The virus was supplied at a titer of 10^7.5^ TCID_50_/ml.

### Ethical approval and experimental animals

All experimental procedures were conducted in compliance with the ethical guidelines approved by the Animal Ethics Committee (AEC) under application number (Vet CU 08072023675). This study was sponsored by the Faculty of Veterinary Medicine, Cairo University. The study utilized different animal models, including suckling mice, weaned mice, lambs, and adult sheep. Suckling mice were used to evaluate the safety of the inactivated RVFV, as described by El-Nimr 1980 (El-Nimr 1980; Faburay et al. 2017), and were monitored for 10 days post-injection. To assess the efficacy and potency of the prepared vaccines, weaned mice aged 22–28 days were used, following the methodology outlined by Eman 1995. For immunogenicity assessment, 21 clinically healthy adult sheep of the local breed, weighing between 35 and 50 kg, were included in the study. These sheep were confirmed seronegative for RVFV antibodies using the serum neutralization test (SNT) and had not been previously vaccinated against RVF. Additionally, seven healthy lambs aged 7 to 10 days were used to evaluate the safety of the vaccines. All animal models were sourced and supplied by VSVRI.

### Adjuvants and co-adjuvants

**Fig. 1:**
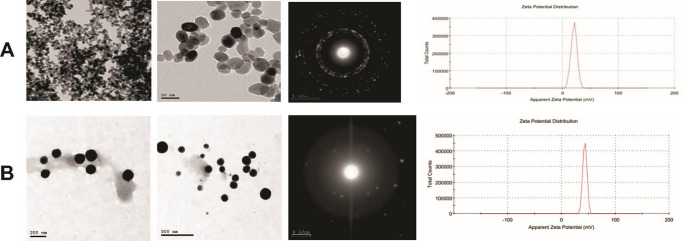
Characterization of zinc oxide nanoparticles (ZnNPs) and selenium nanoparticles (SeNPs). (**A**) Zinc oxide nanoparticles: TEM micrographs at low (left) and high (middle) magnification revealing spherical-like morphology with uniform particle size distribution of 30 ± 5 nm; Selected Area Electron Diffraction (SAED) pattern (right) confirming the crystalline nature; and zeta potential distribution showing a positive surface charge of + 20.6 mV. ZnNPs appear as white-to-yellow colored powder with a molecular weight of 81.408 g/mol. (**B**) Selenium nanoparticles: TEM micrographs at low (left, scale bar 200 nm) and high (middle, scale bar 500 nm) magnification demonstrating spherical morphology with particle sizes less than 200 nm; SAED pattern (right) indicating crystallinity; and zeta potential distribution showing a negative surface charge of + 43.1 mV. SeNPs appear as an orange-colored suspension with a concentration of 500 ppm. The distinct physicochemical properties of these nanoparticles, including their size, surface charge, and morphology, suggest their potential applications as delivery systems or adjuvants in veterinary vaccine formulations

The primary adjuvant used was aluminum hydroxide gel, sourced from VSVRI, at a concentration of 20%. To enhance the immunogenic performance of the vaccines, ZnNPs and SeNPs were utilized as co-adjuvants. Specifically, the dose of ZnNPs incorporated into the vaccine formulations was 200 mg, and the dose of SeNPs was 5 mg. These nanoparticles were provided by Nano-Gate Company and were characterized using transmission electron microscopy (TEM) and zeta potential analysis. TEM revealed that ZnNPs were white-to-yellow spherical particles with an average size of approximately 30 nm and a zeta potential of 20.6 mV. SeNPs, on the other hand, were orange spherical particles with an average size of less than 200 nm and a zeta potential of + 43.1 mV. The detailed characterization of these nanoparticles is presented in Fig. 1.

### Virus titration and inactivation

To provide a comprehensive understanding of our vaccine preparation, the procedures for virus propagation, titration, and inactivation are detailed below.

#### Virus propagation and titration

The virulent ZH-501 strain of RVFV was used in this study, purchased from the Veterinary Serum and Vaccine Research Institute in Abbassia. The viral specimen was cultivated in Hank’s solution and introduced into a fully-grown baby hamster kidney (BHK) cell culture. The cell specimens were cultivated and sustained following the methodology outlined by Macpherson and Stoker 1962. Before experimentation, the cell cultures underwent screening for bovine viral diarrhoea virus (BVDV) and mycoplasma contamination.

For titration, the virus was allowed to adsorb by incubating cell cultures at 37 °C for one hour. Ten microliters of the virus were added to each well, with three wells used for each dilution. After incubation, maintenance media were added, and the cultures were re-incubated at 37 °C. Cytopathic effects (CPE) were monitored daily for 5–7 days. The viral titer was calculated as TCID50 per 0.1 ml of the initial inoculum, using the formula established by Reed and Muench in 1938. The titer of RVFV before inactivation was determined to be 10^7.5^ TCID50/ml.

#### Virus inactivation

The virus was inactivated using binary ethylenimine (BEI), following the methodology described by Girard et al. 1977 and Eman 1995. A virus with a titer of 10^7.5^ TCID50/ml was treated with 1% of 0.1 M BEI in 0.2 N NaOH at 37 °C for 24 h at pH 8.0. Samples were collected at one-hour intervals for up to 24 h. Sodium thiosulfate was added to each sample at a final concentration of 2% to neutralize BEI. Each sample was then inoculated into BHK cell cultures and baby mice to detect residual infectivity. The absence of cytopathic effects (CPE) in cell culture and no deaths in mice at a given interval indicated the optimum time for inactivation. Notably, the inactivation process did not affect the viral titer, which remained at 10^7.5^ TCID50/ml after inactivation.

### Vaccine preparation

Three distinct vaccine formulations were prepared for the present study. The first vaccine batch was prepared using inactivated RVFV, with 20% aluminum hydroxide gel as the primary adjuvant and SeNPs as a co-adjuvant. The second vaccine batch was prepared using inactivated RVFV, with 20% aluminum hydroxide gel as the primary adjuvant and ZnNPs as a co-adjuvant. The third vaccine batch served as a control and was prepared using inactivated RVFV with 20% aluminum hydroxide gel as the sole adjuvant, without any nanoparticles.

### Evaluation of sterility

The sterility of the prepared vaccines was assessed using thioglycollate and soybean casein digest media, as described by El-Nimr 1980. This ensured that the vaccine formulations were free of bacterial, fungal, and mycoplasma contamination.

### Potency testing in mice

The potency of the vaccines was tested in adult mice following the protocol established by Randall et al. and dodd et al. (Dodd et al. 2012). Five-fold serial dilutions of the vaccines, ranging from 1:1 to 1:625, were prepared. Groups of 10 mice were administered a 0.2 ml dose of each dilution intraperitoneally (I/P). A booster dose was given one week later, and the mice were observed for 21 days. Mortality rates were recorded daily, and the effective dose (ED_50_/ml) was calculated using Reed and Muench’s (1938) method (Reed and Muench 1938).

### Safety testing in lambs

The safety of the vaccine formulations was evaluated using seven lambs aged 7 to 10 days, following the methodology outlined by the El-Manzalawy et al. 2014. Each vaccine batch was administered to two lambs at a dose of 10 ml per lamb, while one lamb served as a control. The lambs were monitored for 10 days, and the vaccines were deemed safe if the vaccinated lambs exhibited no clinical abnormalities or adverse reactions during the observation period.

### Vaccination scheme for sheep

A total of 21 sheep were used in the vaccination trial and divided into four groups to evaluate the immunogenicity of the vaccines. Three groups consisted of six animals each and received different vaccine formulations, while the fourth group, serving as the control, consisted of three animals. Group 1 received a 1 ml subcutaneous injection of the vaccine containing inactivated RVFV with aluminum hydroxide gel as an adjuvant and SeNPs as a co-adjuvant. Group 2 received a 1 ml subcutaneous injection of the vaccine containing inactivated RVFV with aluminum hydroxide gel as an adjuvant and ZnNPs as a co-adjuvant. Group 3 received a 1 ml subcutaneous injection of the vaccine containing inactivated RVFV with aluminum hydroxide gel only. Group 4 served as the control and did not receive any vaccine.

### Blood sample collection

Blood samples were collected from the sheep at multiple time points throughout the study. The initial sample was taken on day 0 (pre-vaccination), followed by samples collected on days 1, 3, 4, 7, 10, 14, 21, and 28 post-vaccination. Additionally, blood samples were collected monthly after vaccination until the antibody titers began to decline.

### Evaluation of biochemical parameters

To comprehensively evaluate the physiological impact and safety of the vaccine formulations, and to ensure that the animals’ overall health supports a robust immune response, various biochemical parameters were assessed using serum samples extracted from centrifuged blood at 2000 rpm for 10 min at 4 °C. Total serum protein levels were measured using the Biuret reaction at 540 nm (Reinhold 1953), and serum albumin was determined using the bromocresol green reaction at 630 nm (Doumas et al. 1971). Serum globulin levels were calculated by subtracting the albumin concentration from the total protein concentration. Renal function was evaluated by measuring serum urea and uric acid concentrations, as described by Artiss and Entwistle 1981 and Caraway and Marable 1966 respectively, while serum creatinine levels were determined using the method outlined by Artiss and Entwistle 1981 (Artiss and Entwistle 1981). Liver function was assessed by measuring serum aspartate aminotransferase (AST) and alanine aminotransferase (ALT) levels using the Reitman and Frankel 1957 method (Reitman and Frankel 1957), while total bilirubin was determined following Kaneko et al. 2008.

### Assessment of humoral immune response

To evaluate the humoral immune response, serum samples were subjected to both the SNT and indirect enzyme-linked immunosorbent assay (ELISA). The SNT was conducted in accordance with the previous reports and guidelines (WOAH 2023; Hafez et al. 2025) to detect RVFV-specific neutralizing antibodies. Results were expressed as the reciprocal of the lowest dilution yielding a positive result, with titers above 40 considered protective. Indirect ELISA was performed following the previously described protocol (Voller et al. 1976), and optical density (OD) values were measured at a wavelength of 492 nm using a spectrophotometer. Samples were considered positive if their OD values met or exceeded the cut-off value, calculated as the mean OD of negative control samples plus three times the standard deviation (Kurstak 1985).

### Measurement of IFN-γ

Serum interferon-gamma (IFN-γ) levels were quantified using a double-antibody sandwich ELISA kit (Catalogue No: 201-07-0063, MABTECH^®^, Sweden). The assay was performed according to the manufacturer’s instructions and (Prescott et al. 2002). The OD was measured at a wavelength of 450 nm.

### Statistical analysis

Statistical analyses were conducted using R and SPSS version 20. Group differences were assessed using two-way analysis of variance (ANOVA), followed by Fisher’s Least Significant Difference (LSD) post-hoc test to identify specific pairwise differences. Results are presented as mean ± standard deviation (SD). Statistical significance was defined as a p-value less than 0.05. Distinct letters above data points in the figures denote statistically significant differences among groups.

## Results

This study evaluates the quality, safety, potency, and immunogenicity of inactivated Rift Valley Fever (RVF) vaccines formulated with alum hydroxide gel (ALHV) and enhanced with ZnNPs or SeNPs. The vaccines were rigorously tested for sterility, effectiveness, safety, and their ability to induce strong immune responses.

### Sterility testing of the prepared vaccines

The sterility of the prepared vaccines was confirmed through comprehensive testing, which demonstrated that all formulations were free of mycoplasma, aerobic and anaerobic bacteria, and fungi. These findings validate the production conditions and ensure that the vaccines meet sterility requirements for safe use in subsequent testing. The absence of microbial contaminants underscores the reliability of the manufacturing process and the suitability of the vaccines for preclinical evaluation.

### Potency testing in mice (effective dose 50 (ED50))

The potency of the prepared vaccines was assessed in adult mice by determining the effective dose 50 (ED50). The detailed results of the potency testing, including the number of mice, survival, mortality, and protective ratios for each vaccine dilution, are presented in Table 1. The alum hydroxide-based inactivated vaccine (ALHV) achieved an ED50 of 0.0019/ml, well below the established protective threshold of 0.02/ml, confirming its potency in providing protective immune responses (Randall et al. 1964; Magd et al. 2014). These findings align with established benchmarks for RVF vaccines and support the suitability of the formulations for further testing in larger animal models.

**Table 1:** Potency testing results of inactivated RVF vaccine in mice

Vaccine dilutions	No. of mice	Alive	Dead	Cumulative No.			Protective ratio	Protection %
				Alive	Dead			
1/1	8	7	1	30		1	30/31	96
1/5	8	7	1	23		2	23/25	92
1/25	8	6	2	16		4	16/20	80
1/125	8	5	3	10		7	10/17	58
1/625	8	5	3	5		10	5/15	33

### Safety assessment through clinical examination of vaccinated sheep

To evaluate the safety of the vaccines, clinical assessments were conducted on sheep vaccinated with ALHV, ALHV + ZnNPs, and ALHV + SeNPs. No adverse reactions, unusual symptoms, or post-vaccination complications were observed in any of the groups. Additionally, the mean body temperature of all vaccinated groups remained within normal limits throughout the study period (Table 2), further substantiating the safety of the tested formulations. These findings demonstrate that the vaccines are well-tolerated and safe for use in sheep, a key target population for RVF vaccination.

**Table 2:** The average rectal temperature of individuals administered various inactivated RVF vaccination

Animal group	No. of animals	Zero day	Mean temperature degrees at the following days post-vaccination									
			1st	2nd	3rd	4th	5th	6th	7th	8th	9th	10th
G1	6	39.1	39.3	39.1	38.9	39.2	39.0	39.3	38.9	39.1	39.1	39.1
G2	6	39.3	39.4	39.1	39.0	39.4	39.1	39.2	39.0	39.0	39.3	38.9
G3	6	39.2	39.0	39.1	39.3	39.2	39.1	39.1	39.1	39.2	39.1	39.3
G4	3	39.2	39.1	39.2	39.0	39.3	39.1	39.1	39.2	38.9	39.1	39.1

Group 1: Sheep vaccinated (S/C) with 1ml of (ALHV+SeNPs) as one dose

Group 2: Sheep vaccinated (S/C) with 1ml (ALHV+ZnNPs) as one dose

Group 3: Sheep vaccinated (S/C) with 1ml of (ALHV) as one dose

Group 4: Non-vaccinated sheep (control group)

### Assessment of weight gain in vaccinated sheep

The impact of vaccination on sheep growth performance was assessed by monitoring body weight monthly until the end of the experiment, and (Table 3) shows the average body weight of sheep in each group. Notably, sheep vaccinated with ALHV + ZnNPs exhibited the highest growth rates compared to those vaccinated with ALHV + SeNPs, ALHV alone, and the non-vaccinated control group. These results suggest that ZnNPs not only enhance immunogenicity but may also contribute to improved growth performance, potentially offering additional benefits in livestock vaccination programs.

**Table 3:** Monthly average body weight of sheep vaccinated with RVF vaccines (kg)

Pre-vaccination weight average	Aug	Sep	Oct	Nov	Dec	Jan	Feb	March	Apr	May	June
G1	34	37	41	44	47	51	54	58	61	64	67
G2	35	39	44	48	53	57	61	66	71	76	83
G3	33	36	40	43	47	51	54	57	60	63	66
G4	32	35	38	41	44	47	51	54	57	60	63

Group 1: Sheep vaccinated (S/C) with 1ml of (ALHV+SeNPs) as one dose

Group 2: Sheep vaccinated (S/C) with 1ml of (ALHV+ZnNPs) as one dose

Group 3: Sheep vaccinated (S/C) with 1ml of (ALHV) as one dose

Group 4: Non-vaccinated sheep (control group)

### Serum biochemical analyses

**Fig. 2:**
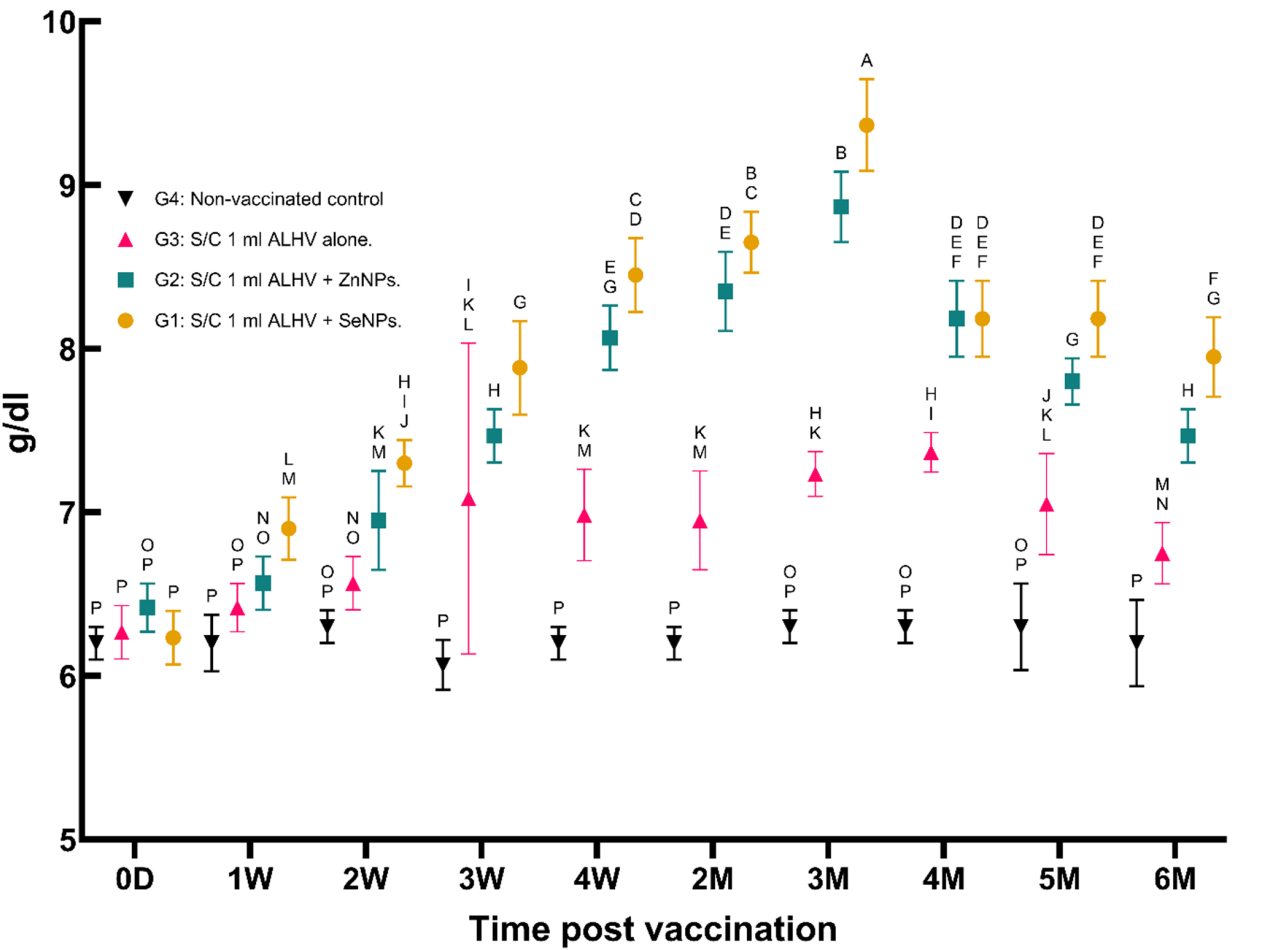
Serum total protein concentration (g/dl) in vaccinated vs. control sheep. Serum total protein concentrations were measured in sheep vaccinated subcutaneously (S/C) with inactivated Rift Valley Fever (RVF) vaccines: Group 1 (ALHV + SeNPs), Group 2 (ALHV + ZnNPs), Group 3 (ALHV alone), and Group 4 (non-vaccinated control). Values were recorded from day 0 up to 6 months post-vaccination. Statistical analyses were performed using two-way ANOVA followed by Fisher’s Least LSD post-hoc test to determine significant differences between groups. Data are presented as mean ± SD. Different letters above data points indicate statistically significant differences between groups at each time point (*p* < 0.05). Vaccinated groups, particularly Groups 1 and 2, showed significant increases in total protein compared to controls, reflecting enhanced immune responses

**Fig. 3:**
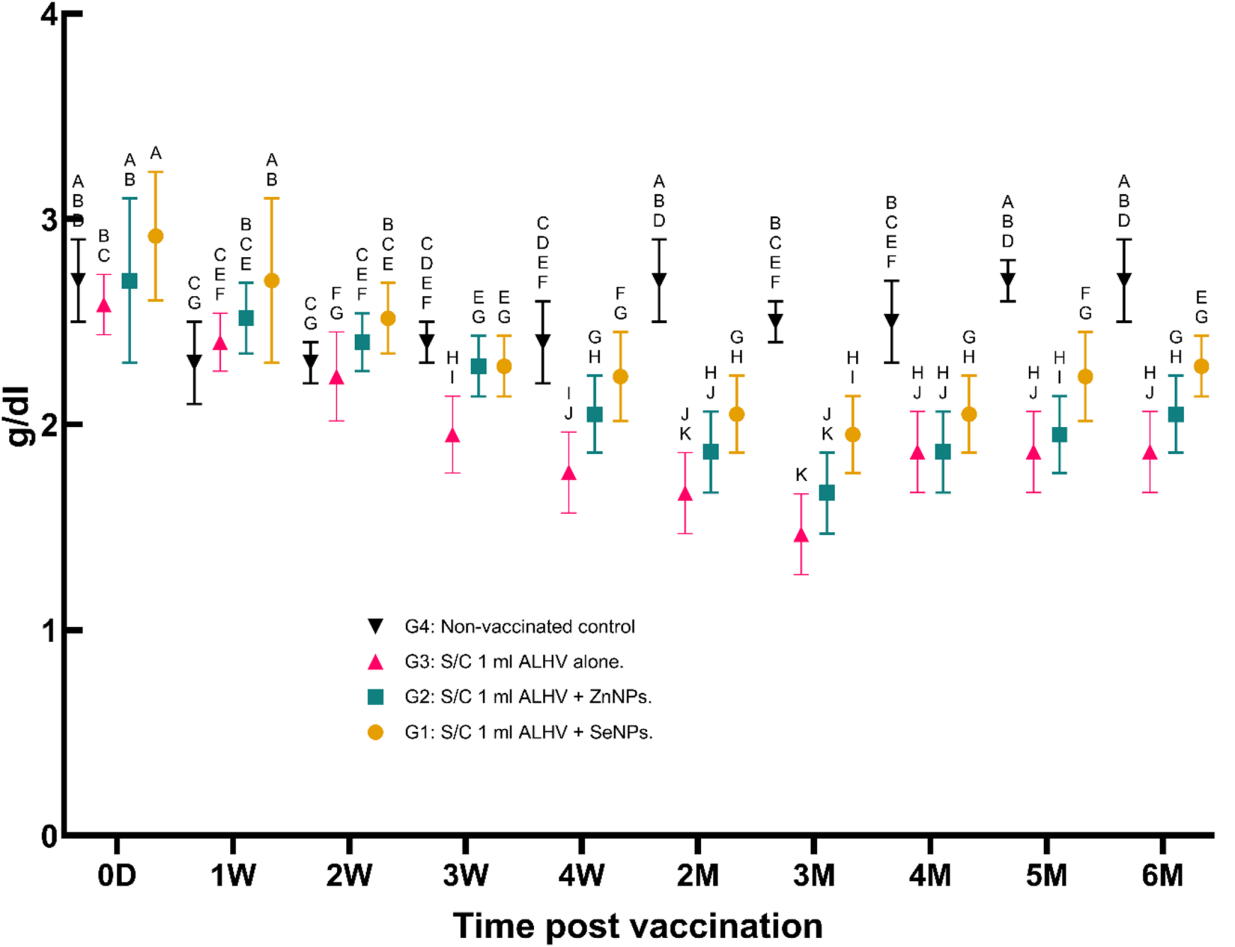
Serum albumin concentration (g/dl) in vaccinated vs. control sheep. Serum albumin concentrations were measured in sheep vaccinated subcutaneously (S/C) with inactivated Rift Valley Fever (RVF) vaccines: Group 1 (ALHV + SeNPs), Group 2 (ALHV + ZnNPs), Group 3 (ALHV alone), and Group 4 (non-vaccinated control). Measurements were taken from day 0 to 6 months post-vaccination. Statistical analyses were performed using two-way ANOVA followed by Fisher’s Least LSD post-hoc test to determine significant differences between groups. Data are presented as mean ± SD. Different letters above data points indicate statistically significant differences between groups at each time point (*p* < 0.05). Albumin levels remained relatively stable across all groups, with no significant adverse effects observed following vaccination

**Fig. 4:**
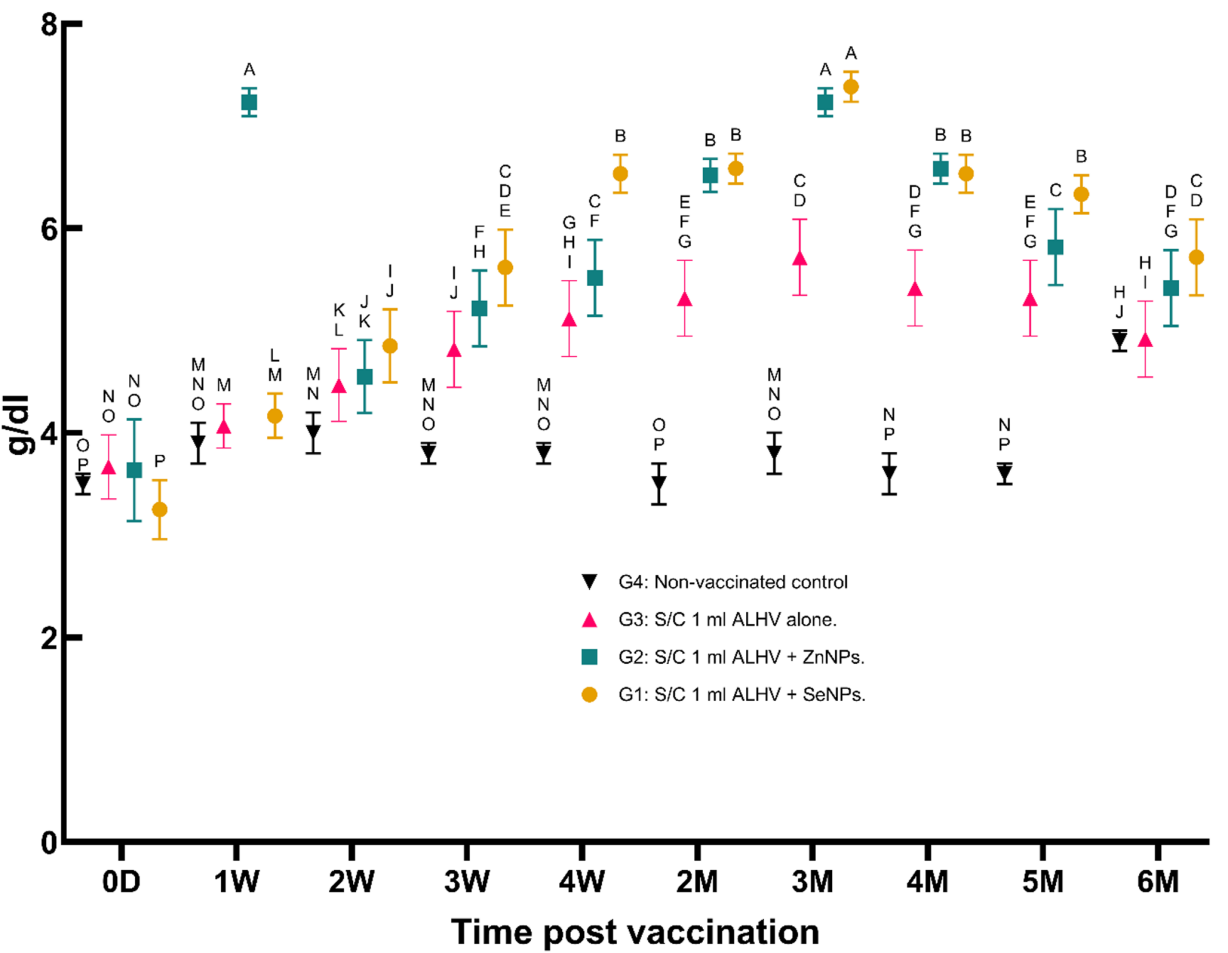
Serum globulin concentration (g/dl) in vaccinated vs. control sheep. Serum globulin concentrations were measured in sheep vaccinated subcutaneously (S/C) with inactivated Rift Valley Fever (RVF) vaccines: Group 1 (ALHV + SeNPs), Group 2 (ALHV + ZnNPs), Group 3 (ALHV alone), and Group 4 (non-vaccinated control). Measurements were taken from day 0 up to 6 months post-vaccination. Statistical analyses were performed using two-way ANOVA followed by Fisher’s Least LSD post-hoc test to determine significant differences between groups. Data are presented as mean ± SD. Different letters above data points indicate statistically significant differences between groups at each time point (*p* < 0.05). Vaccinated groups showed significantly higher globulin levels compared to non-vaccinated controls, with Groups 1 and 2 exhibiting the most pronounced increases over time

**Fig. 5:**
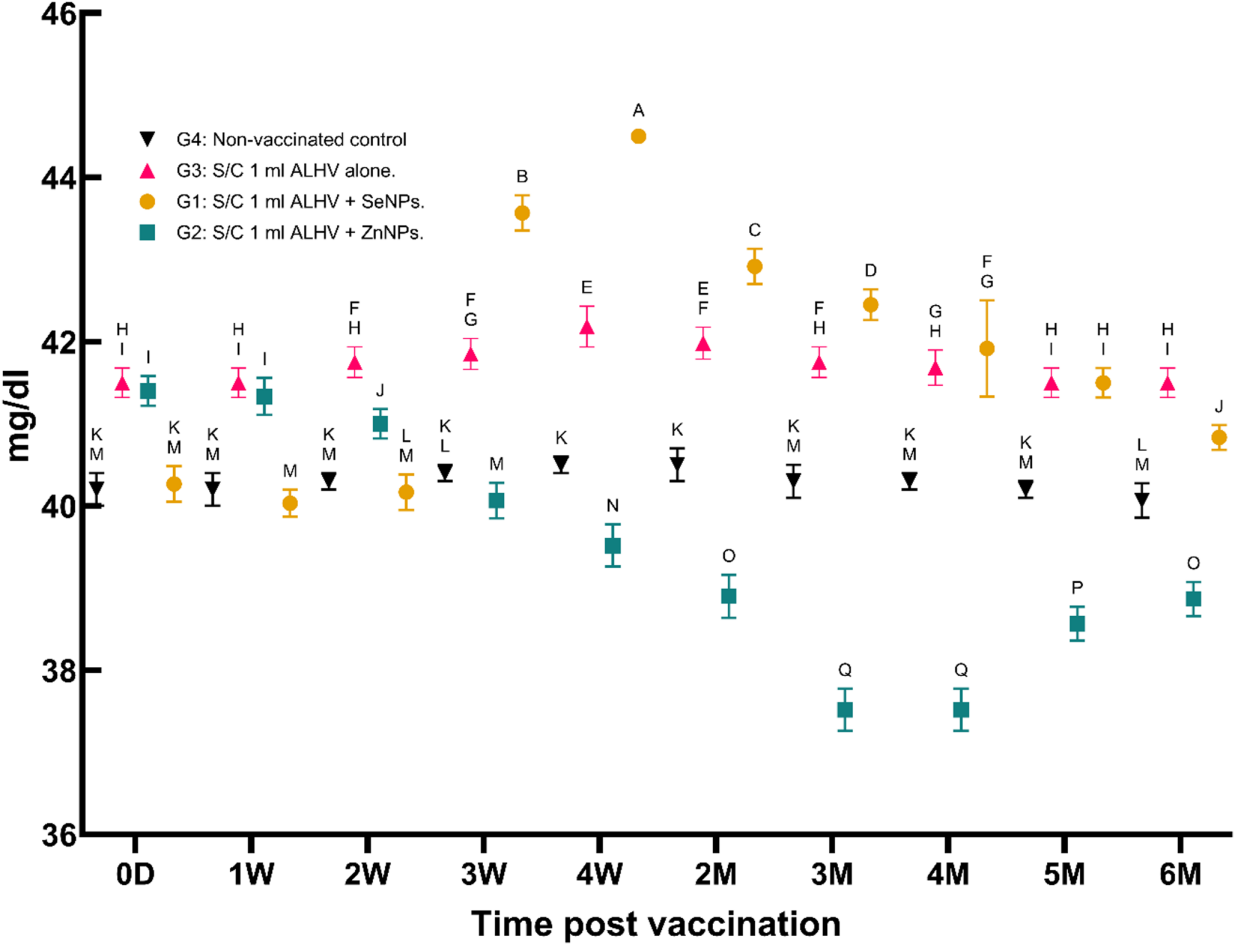
Serum Blood Urea Nitrogen (BUN) concentration (mg/dl) in Vaccinated vs. non-vaccinated control sheep. Serum BUN levels were measured in sheep vaccinated subcutaneously (S/C) with inactivated Rift Valley Fever (RVF) vaccines: Group 1 (ALHV + SeNPs), Group 2 (ALHV + ZnNPs), Group 3 (ALHV alone), and Group 4 (non-vaccinated control). BUN concentrations were monitored from day 0 to 6 months post-vaccination. Statistical analyses were performed using two-way ANOVA followed by Fisher’s Least LSD post-hoc test to determine significant differences between groups. Data are presented as mean ± SD. Different letters above data points indicate statistically significant differences between groups at each time point (*p* < 0.05). Group 1 showed transient elevations in BUN compared to the controls and other groups, while Group 2 exhibited a slight decrease at later time points. Overall, the values remained within the normal physiological range

**Fig. 6:**
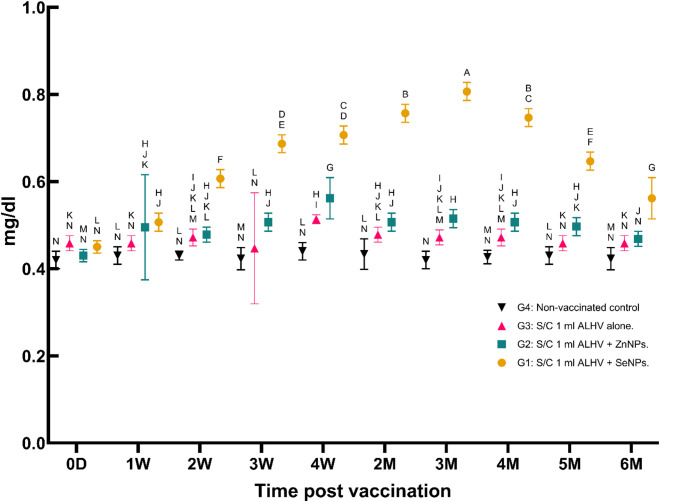
Serum creatinine concentration (mg/dl) in vaccinated vs. non-vaccinated control sheep. Serum creatinine levels were measured in sheep vaccinated subcutaneously (S/C) with inactivated Rift Valley Fever (RVF) vaccines: Group 1 (ALHV + SeNPs), Group 2 (ALHV + ZnNPs), Group 3 (ALHV alone), and Group 4 (non-vaccinated control). Concentrations were monitored from day 0 to 6 months post-vaccination. Statistical analyses were performed using two-way ANOVA followed by Fisher’s Least LSD post-hoc test to determine significant differences between groups. Data are presented as mean ± SD. Different letters above data points denote statistically significant differences between groups at each time point (*p* < 0.05). Group 1 showed a transient increase in creatinine levels compared to controls and other vaccinated groups, but all values remained within normal physiological ranges

**Fig. 7:**
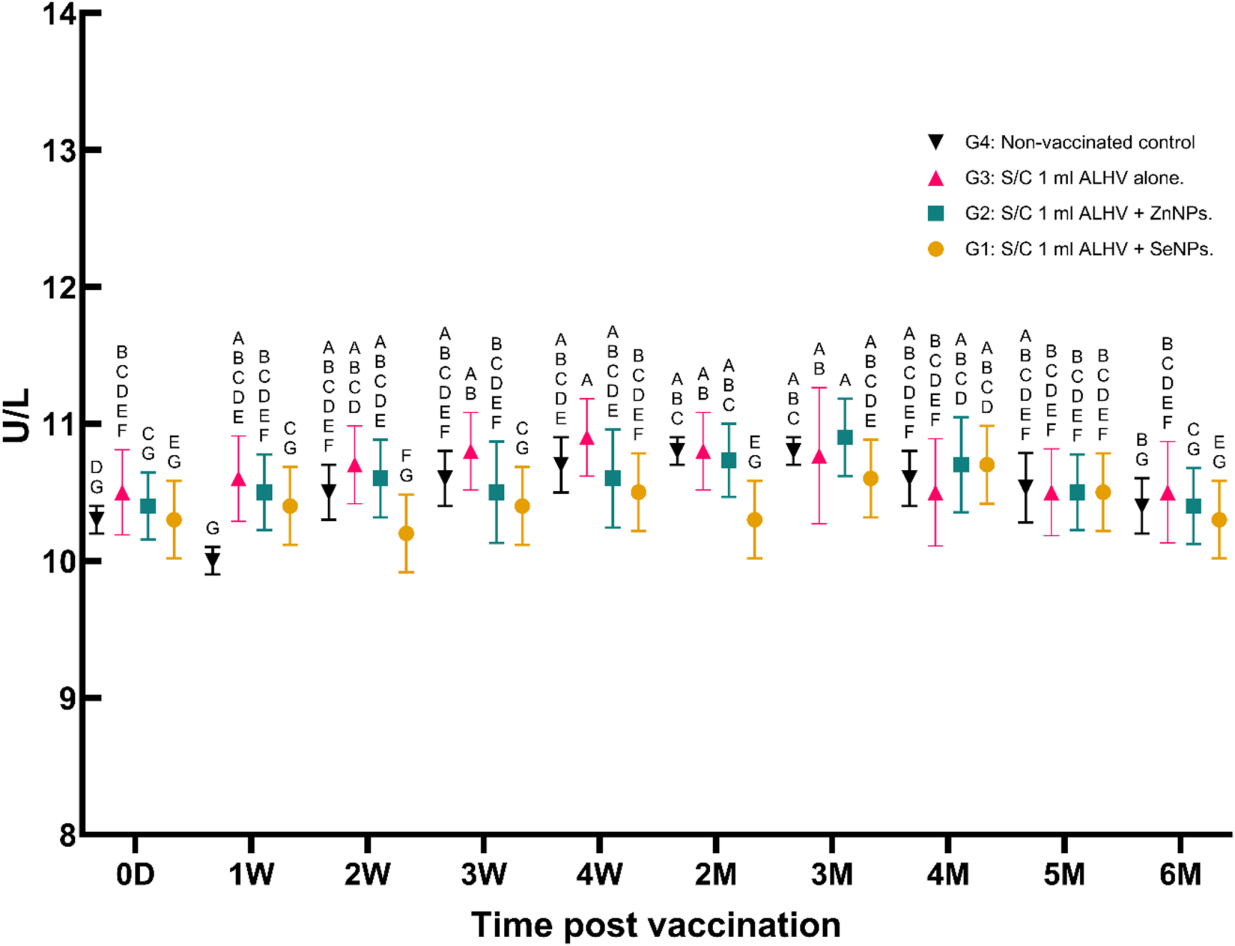
Serum ALT activity (U/L) in sheep: vaccinated vs. non-vaccinated control. Serum alanine aminotransferase (ALT) activity was measured in sheep vaccinated subcutaneously (S/C) with inactivated Rift Valley Fever (RVF) vaccines: Group 1 (ALHV + SeNPs), Group 2 (ALHV + ZnNPs), Group 3 (ALHV alone), and Group 4 (non-vaccinated control). ALT levels were monitored from day 0 to 6 months post-vaccination. Statistical analyses were performed using two-way ANOVA followed by Fisher’s Least LSD post-hoc test to determine significant differences between groups. Data are presented as mean ± SD. Different letters above data points indicate statistically significant differences between groups at each time point (*p* < 0.05). No significant increase in ALT activity was observed in vaccinated groups compared to controls, suggesting no liver toxicity associated with vaccination

**Fig. 8:**
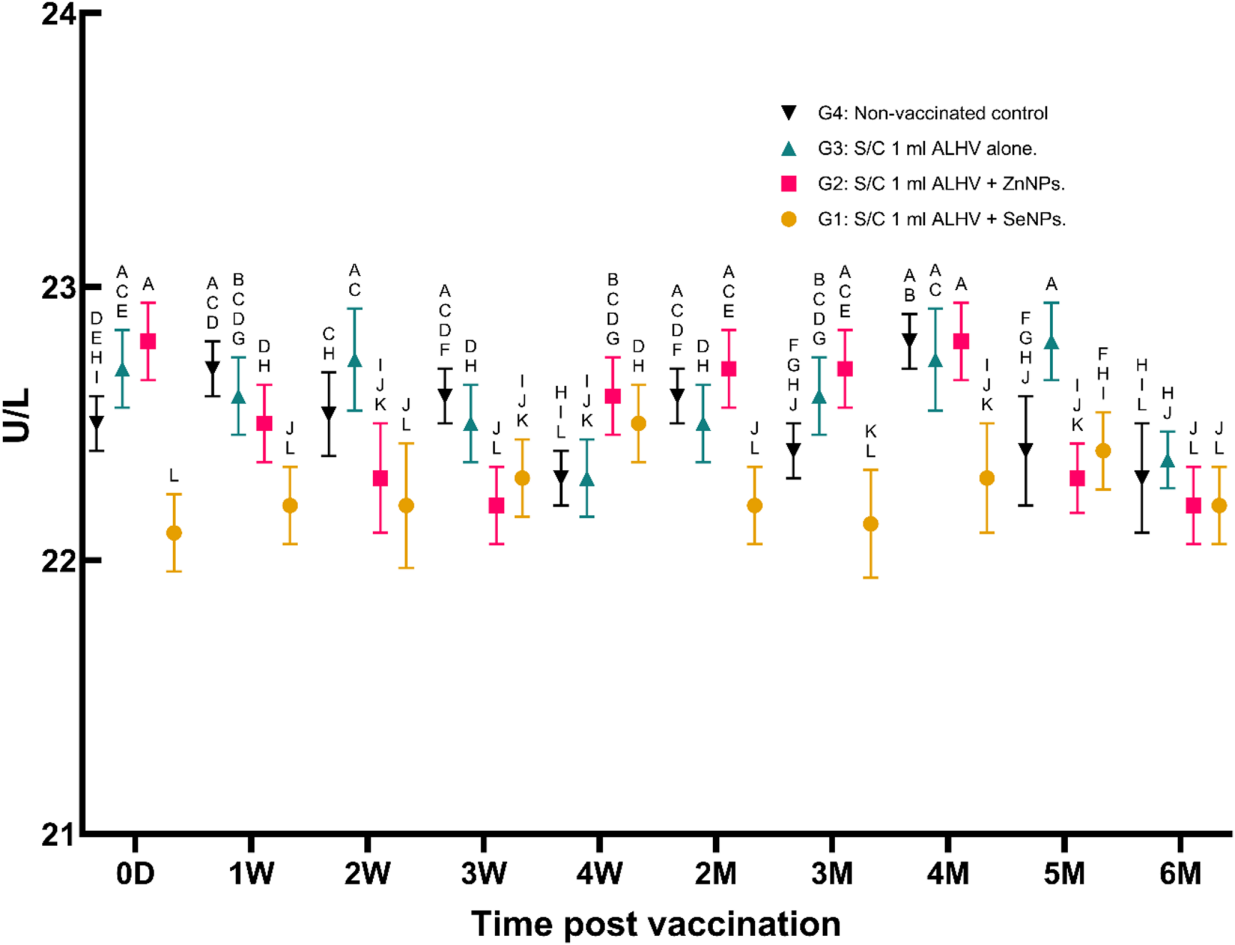
Serum AST activity (U/L) in sheep: vaccinated vs. non-vaccinated control. Serum aspartate aminotransferase (AST) activity was measured in sheep vaccinated subcutaneously (S/C) with inactivated Rift Valley Fever (RVF) vaccines: Group 1 (ALHV + SeNPs), Group 2 (ALHV + ZnNPs), Group 3 (ALHV alone), and Group 4 (non-vaccinated control). AST levels were monitored from day 0 to 6 months post-vaccination. Statistical analyses were performed using two-way ANOVA followed by Fisher’s Least LSD post-hoc test to determine significant differences between groups. Data are presented as mean ± SD. Different letters above data points denote statistically significant differences between groups at each time point (*p* < 0.05). No significant elevation in AST activity was observed in vaccinated groups compared to controls, indicating no adverse hepatic effects from vaccination

Serum biochemical parameters were monitored to evaluate the physiological effects of the vaccine formulations.

i. **Serum total protein concentration (g/dl)**:
  Total protein levels increased significantly in all vaccinated groups, peaking at the third month post-vaccination, before gradually declining. The highest protein levels were observed in the ALHV + SeNPs group, followed by the ALHV + ZnNPs and ALHV alone groups (Fig. 2). This trend indicates enhanced protein synthesis following vaccination, which is consistent with an active immune response.

ii. **Serum albumin concentration (g/dl)**:
  Serum albumin levels decreased significantly during the first week post-vaccination, reaching their lowest levels between the second and third months. These levels gradually returned to baseline by the end of the experiment, with the ALHV + ZnNPs group showing the most stable albumin levels (Fig. 3).

iii. **Serum globulin concentration (g/dl)**:
  Globulin levels showed a significant increase in all vaccinated groups, peaking at the second and third months before gradually declining. The ALHV + ZnNPs group exhibited the highest globulin levels, indicating a strong humoral immune response (Fig. 4).

iv. **Serum Blood Urea Nitrogen (BUN) (mg/dl)**:
  Blood urea nitrogen levels showed slight fluctuations across groups. The ALHV + SeNPs group exhibited a nonsignificant decrease during the first week, followed by a peak at the third month. The ALHV + ZnNPs group showed a mild increase, peaking between the second and third months. Both the ALHV and control groups displayed no significant changes (Fig. 5).

v. **Serum creatinine concentration (mg/dl)**:
  Serum creatinine levels followed a pattern similar to that of urea nitrogen. The ALHV + SeNPs and ALHV + ZnNPs groups exhibited slight fluctuations within the high-normal range, peaking between the second and third months, while the ALHV and non-vaccinated control groups remained stable (Fig. 6).

vi. **Serum ALT and AST activity (U/L)**:
  No significant variations in ALT or AST activity were observed in any of the groups throughout the study period, indicating that the vaccine formulations did not induce hepatotoxicity (Figs. 7 and 8).

### Neutralizing antibody titers

The ability of the vaccines to induce protective immunity was assessed by measuring neutralizing antibody titers.

**Fig. 9:**
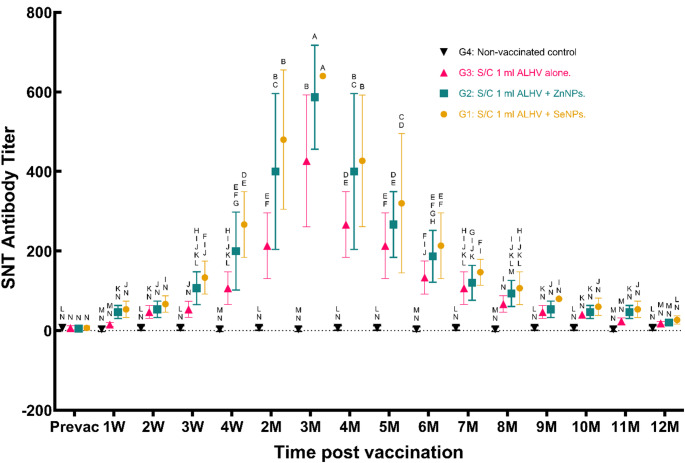
RVF neutralizing antibody titers post-vaccination in sheep. Serum neutralizing antibody titers (SNT) were measured in sheep vaccinated subcutaneously (S/C) with inactivated Rift Valley Fever (RVF) vaccines: Group 1 (ALHV + SeNPs), Group 2 (ALHV + ZnNPs), Group 3 (ALHV alone), and Group 4 (non-vaccinated control). Titers were assessed at baseline (Prevac) and multiple time points up to 12 months post-vaccination. Data are shown as mean ± SD. Different letters above data points indicate significant differences between groups at each time point (*p* < 0.05). Vaccinated groups demonstrated significantly higher neutralizing antibody titers compared to controls, with Groups 1 and 2 showing the strongest and most sustained responses

- In the ALHV + SeNPs group, antibody titers surpassed the protective threshold (> 40) within the first week post-vaccination, peaking at 640 during the third month. By the 11th month, the titers had declined to a non-protective level.
- Similarly, the ALHV + ZnNPs group achieved protective titers (> 40) within the first week, peaking at 586.66 in the third month, before declining to non-protective levels by the 11th month.
- In the ALHV group, titers surpassed the protective threshold in the second week post-vaccination, peaking at 426.6 in the third month and declining to non-protective levels by the 10th month (Fig. 9).
- In summary, these results indicate that both nanoparticle-enhanced vaccines induced a more rapid and robust antibody response compared to ALHV alone, with titers exceeding the protective threshold of 40 within the first week post-vaccination.

### Indirect ELISA results

**Fig. 10:**
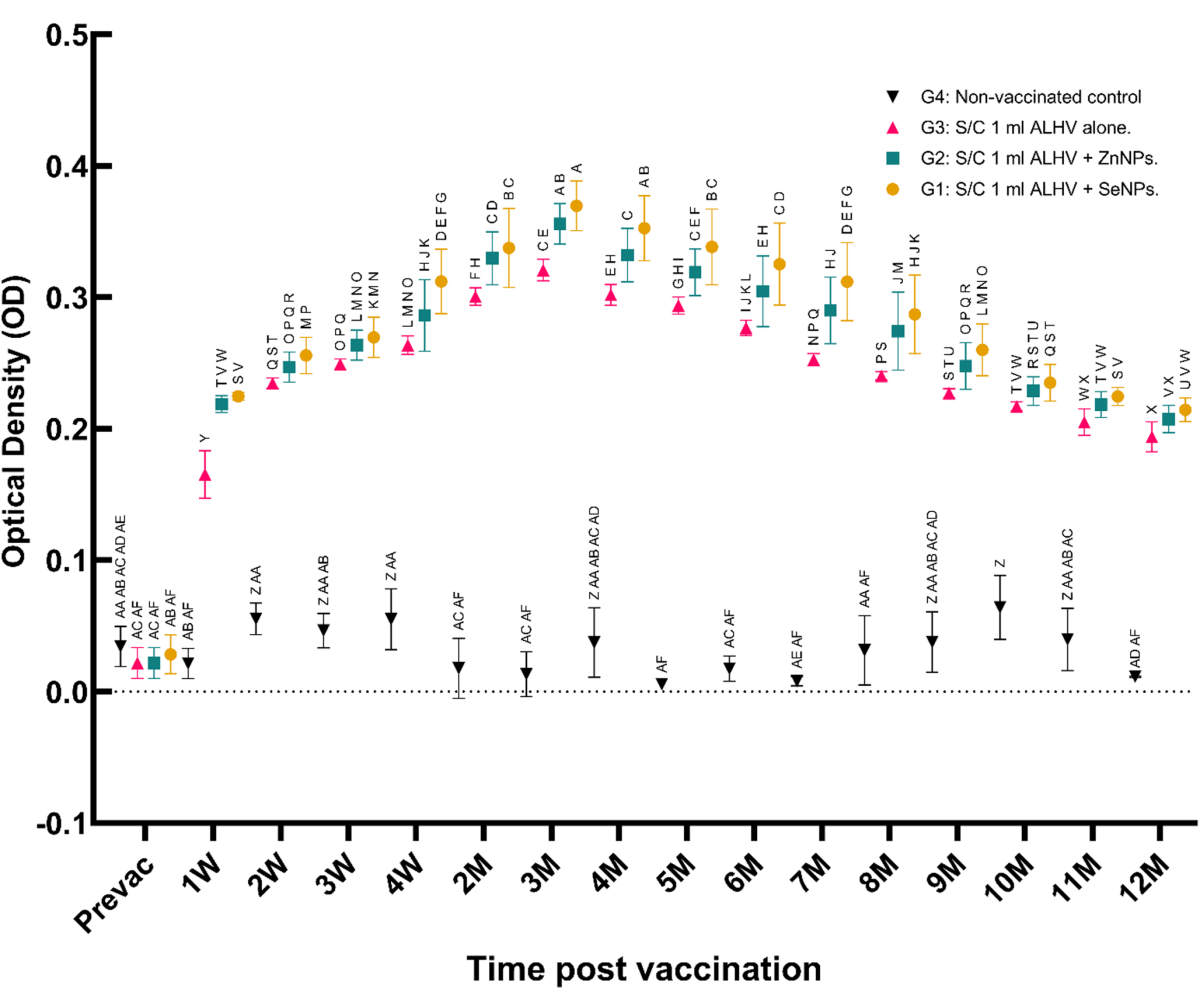
ELISA optical density of sheep immunized with RVF vaccines compared to non-vaccinated controls. Optical density (OD) values measured by ELISA indicate antibody responses in sheep immunized subcutaneously (S/C) with inactivated Rift Valley Fever (RVF) vaccines. Four groups were compared: Group 1 (S/C 1 ml ALHV + SeNPs), Group 2 (S/C 1 ml ALHV + ZnNPs), Group 3 (S/C 1 ml ALHV alone), and Group 4 (non-vaccinated control). Measurements were taken at baseline (Prevac) and at multiple time points up to 12 months post-vaccination. Data are presented as mean ± SD. Different letters above the data points indicate statistically significant differences between groups at each time point (*p* < 0.05). Vaccinated groups showed significantly higher antibody responses compared to controls throughout the study period, with Groups 1 and 2 exhibiting the strongest responses

Indirect ELISA results supported the findings of the neutralization assay. The OD values in the ALHV + SeNPs group became positive in the first week post-vaccination, peaked in the third month, and remained positive until the 11th month. Similarly, the ALHV + ZnNPs group showed positive OD values within the first week, peaking in the third month and remaining positive until the 11th month. In the ALHV group, positive OD values were observed starting in the second week, peaking in the third month, and decreasing to baseline by the 10th month (Fig. 10).

### Serum IFN-γ levels

**Fig. 11:**
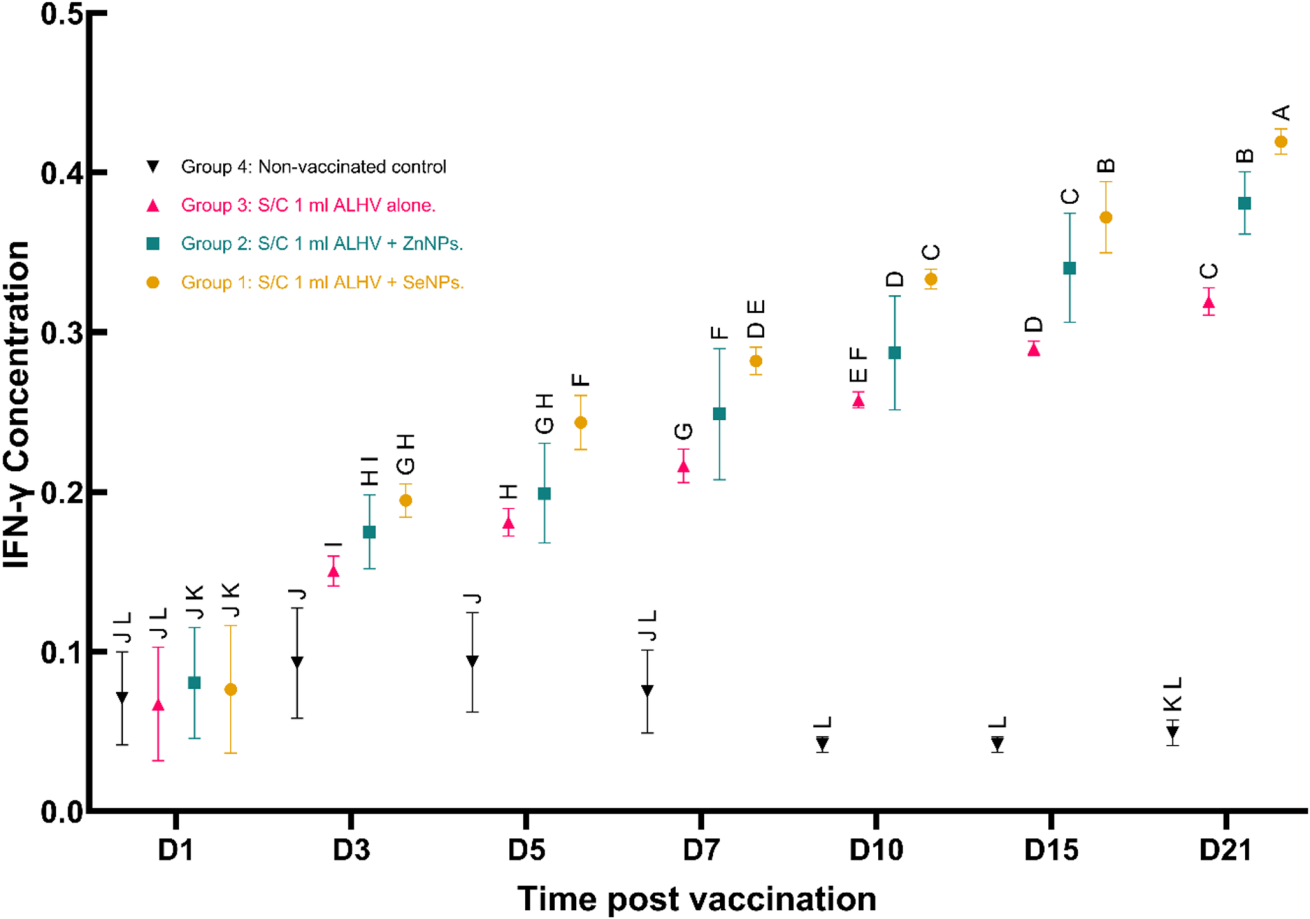
Serum IFN-γ concentrations post-vaccination in sheep. Serum IFN-γ concentrations were measured by ELISA at multiple time points (Days 1, 3, 5, 7, 10, 15, and 21) following vaccination with inactivated Rift Valley Fever (RVF) vaccines. Four groups of sheep were evaluated: Group 1 (S/C 1 ml ALHV + SeNPs), Group 2 (S/C 1 ml ALHV + ZnNPs), Group 3 (S/C 1 ml ALHV alone), and Group 4 (non-vaccinated control). Data represent mean ± standard deviation (SD). Different letters above data points indicate statistically significant differences between groups at each time point (*p* < 0.05). The vaccinated groups exhibited increased IFN-γ levels compared to controls, with Groups 1 and 2 showing enhanced responses relative to Group 3

Serum IFN-γ levels were significantly elevated in all vaccinated groups, indicating robust activation of cell-mediated immunity. The ALHV + SeNPs group exhibited the earliest and highest peak on the fifth day post-vaccination, followed by the ALHV + ZnNPs group on the seventh day and the ALHV group on the 10th day. IFN-γ levels gradually declined in all groups but remained above baseline until the end of the study (Fig. 11).

The results of this study demonstrate that the incorporation of ZnNPs and SeNPs as co-adjuvants in inactivated RVF vaccines enhances vaccine performance by improving immunogenicity, prolonging immune responses, and maintaining safety. Among the tested formulations, the ALHV + ZnNPs vaccine showed the most favorable results in terms of growth performance, antibody titers, and biochemical stability, making it a promising candidate for next-generation RVF vaccine development. These findings support the potential of nanoparticle-enhanced vaccines to address the limitations of existing RVF vaccines and provide effective protection against this devastating zoonotic disease.

## Discussion

### Overview of nanoparticle use in RVF vaccine development

RVFV remains a significant threat to both animal and human health due to its zoonotic nature and mosquito-borne transmission, especially during heavy rainfall and flooding periods (Rostal et al. 2010; Murithi et al. 2011). The virus’s capacity to persist in a dormant state during inter-epizootic phases complicates control efforts, underscoring the critical need for effective vaccination strategies. Vaccination is the most practical approach to protect livestock, which indirectly safeguards human health. However, conventional vaccine adjuvants, such as aluminum hydroxide (alum), have limitations in eliciting strong, durable immunity, motivating exploration of novel adjuvant systems.

Nanoparticles (NPs) have emerged as promising co-adjuvants due to their unique physicochemical properties and intrinsic immunostimulatory effects. They facilitate enhanced antigen delivery via improved bioavailability and controlled release, leading to rapid immune activation and sustained humoral and cellular responses while maintaining safety (Barnowski et al. 2019; Priyanka et al. 2023). This study demonstrates that the incorporation of ZnNPs and SeNPs as co-adjuvants in alum hydroxide-based inactivated RVF vaccines significantly enhances both humoral and cellular immune responses. The nanoparticles not only accelerate the onset of protective immunity but also extend the duration of antibody-mediated protection, thereby addressing the limitations of conventional vaccine formulations. This aligns with broader scientific evidence showing nanoparticles amplify immune responses through targeted immunomodulation.

### Physicochemical characterization of nanoparticles

Characterization revealed ZnNPs as spherical particles approximately 30 nm in diameter, while SeNPs were larger but also spherical, below 200 nm. Their zeta potentials (20.6 mV for ZnNPs and + 43.1 mV for SeNPs) indicate good stability and compatibility for biomedical use. The small size of ZnNPs favors efficient cellular uptake and antigen delivery, whereas SeNPs’ larger size supports prolonged immune stimulation (Bai et al. 2017; Priyanka et al. 2021). These distinct properties support their roles as effective vaccine co-adjuvants.

### Clinical safety and growth performance in vaccinated sheep

Following the physicochemical characterization of the nanoparticles, we next assessed their clinical safety and impact on growth performance in vaccinated sheep. Safety is paramount for veterinary vaccines. Across all vaccinated groups “ALHV alone, ALHV + ZnNPs, and ALHV + SeNPs” no adverse clinical signs or pyrexia were observed; body temperatures remained normal, affirming safety. Notably, sheep receiving “ALHV + ZnNPs” showed significantly increased body weight compared to other groups, consistent with previous studies linking ZnNPs to improved growth performance in lambs (Abd elgayed et al. 2022). This superior growth likely reflects ZnNPs’ dual role as immunomodulators and micronutrient supplements optimizing metabolic functions. SeNPs also enhanced weight gain but to a lesser extent, paralleling findings in foot-and-mouth disease vaccine studies (Hamzah and Dawood 2024). These growth benefits may relate to the nanoparticles’ influence on hepatic fatty acid metabolism, energy production, and enzyme regulation. To ensure that this favorable in vivo profile is matched by manufacturing quality, we next assessed core product attributes—sterility, potency, and physical stability.

### Sterility, potency, and vaccine quality

Sterility testing confirmed absence of bacterial, mycoplasma, and fungal contamination in all vaccine preparations (WOAH 2023). Potency assays showed that the effective dose (ED50) for all formulations was 0.0019/ml—well below the protective threshold of 0.02/ml (Dodd et al. 2012). Vaccine emulsions maintained physical stability throughout the study without phase separation. These results align with prior reports of stable RVF vaccine formulations (Jang et al. 2011), confirming the robustness and quality of the nanoparticle-enhanced vaccines.

### Immunological mechanisms and safety profile

Safety evaluations revealed no adverse clinical or biochemical effects associated with either nanoparticle-enhanced formulation. Liver and kidney function markers remained within the normal range, confirming the biocompatibility of ZnNPs and SeNPs in sheep. These results are consistent with previous work demonstrating improved safety profiles for nanoparticle adjuvants versus traditional alternatives (Jampilek et al. 2019).

### Impact of nanoparticles on serum biochemical parameters

Sheep vaccinated with “ALHV + SeNPs” showed significant increases in total protein and globulin levels, indicating enhanced immune responses, consistent with earlier studies on selenium’s immunomodulatory effects (Hussein et al. 2019). SeNPs may modulate antioxidant activity and gene expression, thereby contributing to these effects.

Similarly, “ALHV + ZnNPs” induced elevated serum protein and globulin levels, reflecting enhanced protein synthesis (Mohamed et al. 2015). The elevated globulin levels in the ZnNP group indicate a strong humoral response that favors Th2-type immunity, which is beneficial for neutralizing the virus. Slight increases in urea and creatinine levels remained within the high-normal limits, indicating no detrimental kidney effects. Liver enzymes (ALT, AST) stayed within normal ranges across all groups, further confirming safety.

### Immunogenicity and antibody responses

Protective neutralizing antibody titers (> 40) were attained within 3 days post-vaccination in the “ALHV + SeNPs” group and within 4 days in the “ALHV + ZnNPs” group, much faster than the 2-week onset in ALHV alone.

The rapid activation of immune responses parallels observations from recent studies on lipid nanoparticle adjuvants, which induce earlier and stronger antibody responses in viral vaccines (Cui et al. 2024; Dai et al. 2024), suggesting a generalizable mechanism across nanoparticle types.

Peak titers reached 640 (SeNPs) and 586.66 (ZnNPs) at three months, maintaining protection until month 11. In contrast, ALHV-alone titers peaked at 426.6 but declined below the protective levels by month 10. ELISA confirmed these trends, with the strongest sustained humoral immunity observed in the nanoparticle groups.

These findings demonstrate the capacity of nanoparticles to enhance both the magnitude and duration of antibody-mediated protection.

### Cellular immunity and IFN-γ responses

Cell-mediated immunity was also enhanced by the nanoparticle formulations. Serum IFN-γ levels—a critical antiviral cytokine—increased significantly early post-vaccination: peaking on day 5 for ALHV + SeNPs, day 7 for “ALHV + ZnNPs”, and day 10 for ALHV alone. IFN-γ levels remained elevated above the baseline until the end of the study, consistent with effective cellular immune activation (Abd El Rahman et al. 2020).

The distinctive immunomodulatory role of SeNPs was evidenced by their earliest and highest IFN-γ peak on day five post-vaccination. This aligns with prior research showing selenium’s ability to activate T cells, NK cells, and other immune pathways (Yazdi et al. 2015), emphasizing SeNPs’ capacity to stimulate robust innate and adaptive immune responses.

The combined data suggest that nanoparticle adjuvants promote a balanced Th1/Th2 immune response, characterized by elevated IFN-γ and strong neutralizing antibodies. This balanced profile resembles that reported for calcium phosphate nanoparticles known to induce more comprehensive immunity than aluminum salts alone (Lin et al. 2017). The early IFN-γ response may involve NLRP3 inflammasome activation, a key pathway mediating nanoparticle-induced immune enhancement, although this was not directly measured in this study (Ahmed et al. 2024).

### Advantages over conventional RVF vaccines

The critical advancements demonstrated in this study include: (i) accelerated onset of protective immunity, with antibody titers exceeding 40 achieved within one week post-vaccination in both the “ALHV + ZnNPs” and “ALHV + SeNPs” groups, compared to two weeks in the ALHV-alone group; (ii) extended duration of protective immunity to 11 months for vaccines containing nanoparticles versus 10 months for ALHV alone; and (iii) superior growth performance observed in the ZnNP group, enhancing both animal health and productivity.

These improvements have practical significance for vaccination campaigns in endemic areas by potentially reducing booster frequency and associated costs.

### Limitations of the study

Despite these promising results, several limitations must be acknowledged: (i) the precise immunological mechanisms involved, such as activation of the NLRP3 inflammasome or Toll-like receptor pathways, remain unverified; (ii) evaluation was limited to sheep, leaving translational potential to other hosts, including cattle, camels, and humans, unexplored; (iii) no challenge experiments were conducted to confirm sterile immunity; (iv) potential antigenic competition between nanoparticles and viral antigens was not investigated; (v) the durability of growth-promoting effects beyond 11 months and their correlation with metabolic markers remain unknown; (vi) long-term toxicity concerns related to zinc accumulation were not addressed; (vii) fixed nanoparticle concentrations were used without dose or size optimization; (viii) mucosal immunity, including IgA responses critical for blocking vector transmission, was not assessed; (ix) safety profiling lacked histopathological analysis and evaluation in pregnant animals, and (x) and practical challenges related to broader clinical application and large-scale implementation, such as regulatory hurdles and the cost-effectiveness and scalability of nanoparticle production, were not fully addressed. Addressing these gaps is essential for clinical translation and vaccine development.

### Future directions and translational potential

Future research should focus on the following key areas: (i) clarifying the molecular mechanisms underlying the adjuvant effects of ZnNPs and SeNPs, including their impact on antigen processing, pattern recognition receptor activation, memory cell generation, and inflammasome pathways; (ii) optimizing the physicochemical properties of nanoparticles for maximal efficacy; (iii) extending studies to other susceptible species within a One Health framework, such as camels, cattle, and potentially humans; (iv) investigating mucosal immunity induction to block vector-borne transmission; (v) conducting challenge studies to confirm protective efficacy; (vi) evaluating long-term safety, including histopathology and reproductive safety assessments; and (vii) exploring dose-response relationships to support scalable vaccine production.

## Conclusion

In this study, we successfully developed and evaluated inactivated RVF vaccines formulated with alum hydroxide gel and enhanced with ZnNPs and SeNPs as co-adjuvants. This study highlights several key advantages of nanoparticle-enhanced formulations: (i) they demonstrated excellent safety profiles, with no adverse clinical signs observed in vaccinated sheep, (ii) they elicited robust humoral and cellular immune responses, (iii) they significantly extended the duration of protective immunity, (iv) they improved growth performance in vaccinated sheep, particularly in the ZnNPs group, and (v) they maintained consistency and sterility throughout the study, ensuring the quality of the vaccine formulations.

While the outcomes are promising, two limitations need to be addressed: (i) the cost and scalability of nanoparticle production must be carefully evaluated for large-scale implementation, and (ii) further field studies are warranted to validate the efficacy of these formulations under real-world conditions and across diverse livestock environments.

The use of ZnNPs and SeNPs as co-adjuvants offers a transformative approach to vaccine development. These nanoparticles enhance both humoral and cellular immunity while maintaining safety and stability, making them valuable tools in the fight against RVF. Furthermore, their adaptability suggests potential applications in vaccines for other zoonotic and infectious diseases, making this approach broadly applicable across veterinary and public health domains.

Based on the promising results of this study, nanoparticle-enhanced RVF vaccines have the potential to play a pivotal role in mitigating the burden of this disease in livestock populations. By improving vaccine efficacy and extending protective immunity, these formulations have the potential to safeguard animal health, enhance livestock productivity, and indirectly protect human populations from zoonotic transmission.

Future research should focus on optimizing nanoparticle dosages, improving cost-effectiveness, and expanding the application of this technology to address emerging infectious disease threats.

## Data Availability

All data are presented within the manuscript.
